# Psychometric properties of Arabic-translated-related quality of life scales for people with parkinson disease: a scoping review

**DOI:** 10.1186/s12889-024-20002-0

**Published:** 2024-09-14

**Authors:** Chiraz Azaiez, Naser Chalghaf, Amayra Tannoubi, Noomen Guelmami, Medina Srem-Sai, Frank Quansah, John Elvis Hagan, Heifa Sneni, Ghada Boussayala, Imane Ghalmi, Mazin Inhaier Lami, Nicola Luigi Bragazzi, Stephane Mandigout, Choukri ben Ayed, Fairouz Azaiez

**Affiliations:** 1 Sociological Research Group on Contemporary Societies (GRESCO), University of Limoges, BP 23204, Limoges, 87032 France; 2 Institute of Sport and Physical Education of Gafsa, University of Gafsa, Gafsa, 2100 Tunisia; 3 Higher Institute of Sport and Physical Education of Sfax, University of Sfax, Sfax, 3047 Tunisia; 4 Ostgraduate School of Public Health, Department of Health Sciences (DISSAL), University of Genoa, Genoa, 16126 Italy; 5 Higher Institute of Sport and Physical Education of Kef, University of Jendouba, Kef, 7100 Tunisia; 6 Department of Mathematics and Statistics, York University, Toronto, ON 4700 Canada; 7 Department of Health, Physical Education, Recreation and Sports, University of Education, P.O. Box 25, Winneba, Ghana; 8 Department of Educational Foundations, University of Education, P.O. Box 25, Winneba, Winneba, Ghana; 9 Department of Health, Physical Education and Recreation, University of Cape Coast, PMB TF0494, Cape Coast, Ghana; 10 Neurocognition and Action-Biomechanics-Research Group, Faculty of Psychology and Sports Science, Bielefeld University, Postfach 10 01 31, 33501 Bielefeld, Germany; 11 Université de Mohamed Cherif Messadia, Souk Ahras, 41000 Algeria; 12 College of Physical Education and Sports Sciences, University of Wasit, 52001, Wasit, Iraq; 13 University of Limoges, HAVAE, Limoges, 20217, F-87000 UR France

**Keywords:** Health-related quality of life, Parkinson’s disease, Quality of life, Scale validation

## Abstract

**Background:**

Parkinson’s disease (PD) substantially contributes to poor functional outcomes, loss in productivity, and poor health-related quality of life (HRQoL). Despite the existence of various scales, there is a notable gap in existing HRQoL reviews with regard to the availability of Arabic validated scales. As a response to this gap, the aim of our scoping review is to identify validated scales, focusing on their psychometric validation procedures, to contribute valuable insights to the understanding of HRQoL among the Arabic-speaking people with PD.

**Methods:**

A scoping review was conducted at the end of December 2022, using the Medline and Embase databases. The focus of this review was on examining the psychometric properties and validation procedures of included scales. Articles were included in the full-text screening process if they focused on people with PD of any age, included a scale measuring HRQoL in Arabic, and were written in English, French, or Arabic.

**Results:**

After applying inclusion/exclusion criteria, 10 studies were selected to analyze HRQoL scales validated in people with PD. However, the PDQ-39 is the only HRQol PD specific scales validated in the Arabic language. Five studies validated in people with PD were identified in the context of instrument validation (3 generic, 1 specific validated in 2 studies).

**Conclusion:**

There are several HRQoL measurement scales for people with PD. However, only one specific HRQoL instrument has been validated in Arabic for people with PD. For the remaining instruments identified they were just used in people with PD without being validated in this population.

**Supplementary Information:**

The online version contains supplementary material available at 10.1186/s12889-024-20002-0.

## Introduction

Parkinson’s disease (PD) is an age-related disorder typically emerging between 55 and 65 years old, affecting 1% of those over 60, escalating to 3.5% at ages 85–89 years, with a higher male prevalence [1, 2]. Over 10 million globally have PD, expected to reach 12 million by 2030 [3]. In Arab countries, PD prevalence surged from 27 to 43 per 100,000 in 1990 to 82.6 per 100,000 people in 2019 [4–8]. Symptoms include motor and non-motor indicators, where the former pertains to movement and the latter doesn’t.

Motor symptoms like bradykinesia, hypokinesia, and rigidity define parkinsonism, crucial for diagnosis [9, 10]. Non-motor symptoms, appearing early, significantly contribute to overall symptomatology, encompassing depression, anxiety, cognitive impairment, and more [11–13]. These symptoms, due to autonomic dysfunctions, mood changes, and cognitive deterioration, complicate disease progression [11–13].

PD is challenging to assess initially, with patients often unaware or reluctant to report their symptoms [14, 15]. Patient’s perception of health aids diagnosis and monitoring [16]. People with PD, especially those with cognitive decline, may die 2.4 years earlier than the general population, exacerbated by fall risks, totalling 5 years earlier [17, 18]. This usually occurs 7 to 15 years into the disease, significantly impacting their quality of life (QoL) [19–21].

Understanding PD requires examining genetics, environment, and lifestyle factors [22–24]. Early biomarkers can aid diagnosis earlier and enhance the care, underscoring the need for improved medical procedures and support networks [23, 25]. As PD progresses, tracking QoL becomes crucial, as it declines with disease severity [26, 27]. Overall, PD reduces life expectancy and substantially affects QoL. Monitoring QoL throughout the disease trajectory is vital, reflecting disease progression [26, 27].

Since the 1980s, health-related quality of life (HRQoL) has been regarded as a subjective concept [28]. Defined by the European Medicines Agency and the Food and Drug Administration as the patient’s perception of how his or her condition and treatment(s) affect everyday activities as well as psychological, physical, and social interaction and well-being [28–30]. HRQoL encompasses various variables, including life satisfaction, economic well-being, activities of daily living, personal well-being, and mental and physical health [31, 32]. Particularly in neurological disorders like PD, HRQoL assessment serves as a scale to evaluate functional impacts of medical conditions and treatment outcomes [33, 34]. Scales used to measure HRQoL can be generic or condition-specific, with [35]. Regulators are increasingly inclined to standardize HRQoL scales internationally and ensure pertinent questions are asked, as technology advancements improve HRQoL data reliability [36, 37].

Arabic ranks as the fifth most widely spoken language globally, boasting approximately 420 million native speakers, which constitutes around 5% of the world’s population. It serves as the official language in 27 countries spanning the Middle East and North Africa (MENA) region [38]. It is essential that measures are culturally relevant, ensuring that a scale is accessible, reliable and valid within each specific cultural context [39, 40]. It improves access to healthcare and research opportunities for Arabic speakers and supports advances in research and clinical practice. It also improves patient outcomes through accurate assessment of QoL, symptom severity and treatment effectiveness [40, 41].

While previous systematic reviews by Martinez et al. (2012) and Berardi et al. (2022) evaluated HRQoL in people with PD, they did not include information on HRQoL scales validated in the Arabic population [42, 43]. Therefore, this scoping review aims to fill this gap by identifying HRQoL scales validated in Arabic in people with PD, assessing their psychometric properties and validation process, offering valuable insights into HRQoL assessment in this population.

## Materials and methods

The review followed the guidelines outlined in the PRISMA Extension for Scoping Reviews Checklist and Explanation, published by Tricco and colleagues, in 2018 [44]. The methodology was divided into three main steps.

First, studies that met the predefined inclusion criteria were identified through a study selection process. This was followed by data extraction and synthesis of the evidence, starting with a qualitative analysis. The qualitative analysis focused on the general characteristics of the scales and the assessment of their quality parameters. Finally, a quantitative analysis was conducted to further synthesise the evidence. To ensure a thorough evaluation, a quality criteria rating grid was developed to assess the scales used to measure HRQoL in people with PD, focusing on two key areas: scale development and its psychometric evaluation.

### Study selection and identification of the source of evidence

A scoping review was conducted in late December 2019 and updated late December 2022, in Medline and Embase databases via PubMed and ScienceDirect. A search algorithm was implemented in both databases to include relevant article (see supplementary Table 1).

Inclusion criteria encompassed all people with PD, with no age restriction, and studies that used scales measuring QoL or HRQoL in Arabic. All study designs were eligible. Exclusion criteria included studies not involving humans, those not addressing Patient-Reported Outcomes (PRO), QoL, or HRQoL indicators, studies that did not use scales measuring PRO, QoL, or HRQoL in people with PD, and duplicate studies.

The selection process involved two phases: title and abstract screening, followed by full-text screening.The title and abstract screening phase was conducted independently by two reviewers, including all articles related to PD and QoL or HRQoL scales, written in English, French, or Arabic. During the full-text screening, the same inclusion and exclusion criteria were applied, with an additional requirement: only studies that included generic or specific HRQoL scales that were either validated or used in Arabic-speaking people with PD were included, without any age restrictions. No limitations were imposed on the publication date or geographical region. Any discrepancies between the reviewers were resolved through discussion, and by consulting a third reviewer.

Additionally, complementary research was conducted on Google Scholar using potentially relevant references found during the full-text screening of included articles. For example, if a scale version was used in an article but the original validation study was not identified during the full-text screening, a search for the validation article was conducted on Google Scholar.

### Data extraction and synthesis of the evidences: qualitative analysis

For the qualitative analysis, a study was included if it is about HRQol scale validated or used for people with PD. Scales such as the Nottingham Health Profile (NHP), the Scales for Outcomes in Parkinson’s Disease - Autonomic Dysfunction (SCOPA-AUT) and the Parkinson’s Well-Being Map were excluded because their primary purpose is not to assess HRQoL, but rather assessed general health or well-being, or only one dimension of HRQoL. Other reasons for exclusion included: only abstracts published, studies not using an HRQoL scale, or people with PD.

The simplified scoping grid identified HRQoL scales (see supplementary Table 2). This included general scale characteristics (generic/specific, number of dimensions, number of items, number of patients included in the study, scoring system of the scale and number of languages). In addition, 7 quality parameters were also included, with the use of the same 4-point rating scale: ++ high; + medium; - low; 0 not reported in (see supplementary Table 2). The grid has been used by Gazzard and colleagues, in 2021 for Glaucoma Patients [45] and was adapted in our study taking into account the recommendations of the PRISMA-ScR checklist [44].

### Data extraction and synthesis of the evidences: quantitative analysis

A quality criteria rating grid (see supplementary Table 3) was developed to assess scales used to measure HRQoL in people with PD. The design of the criteria rating grid was developed by Gazzard and colleague, in 2021 [45] and includes two main areas (development of the scale and psychometric evaluation). It should be noted that the methodological standards for how each item should be scored were detailed in the grid for the rater who was scoring every included scale. This methodology that was initially developed for Glaucoma Patients was adapted for use in people with PD.

All included studies were imported into this grid. The first step of the selection process was to remove all duplicates. Two reviewers independently assessed titles and the abstracts of collected publications for possible inclusion in the study. After that, a full-text screening was separately conducted to identify existing HRQoL scales used or developer in people with PD. Then, the parameters used for the assessment of each of the HRQoL scales in the included publications and the results were summarised in a table using a 4-point rating scale: ++ high; + medium; - low; 0 not reported. Disagreements were resolved by consensus in the presence of a third reviewer.

The completed quality criteria grid for the included HRQoL scales was further quantitatively assessed by scoring the 21 qualities assessed in Parts I and II available in (the supplementary Table 3). In the results, the 4-point rating scale (++, +, -, 0) was numerically converted: ++ was assigned a score of 2, + was assigned a score of 1, - was converted to -1, and 0 retained its numerical value, resulting in a maximum total score of 42. This conversion was necessary because not all parameters allowed a maximum score of 2; for example, measurement error was scored on a scale from − 1 to 2. and includes only qualitative descriptive. It is important to note that this method has already been used in a study by Gazzard and colleagues, in 2021 [45] and that we have re-adapted it for use in our study.

## Results

### Identification and selection of source of evidence

**Fig. 1:**
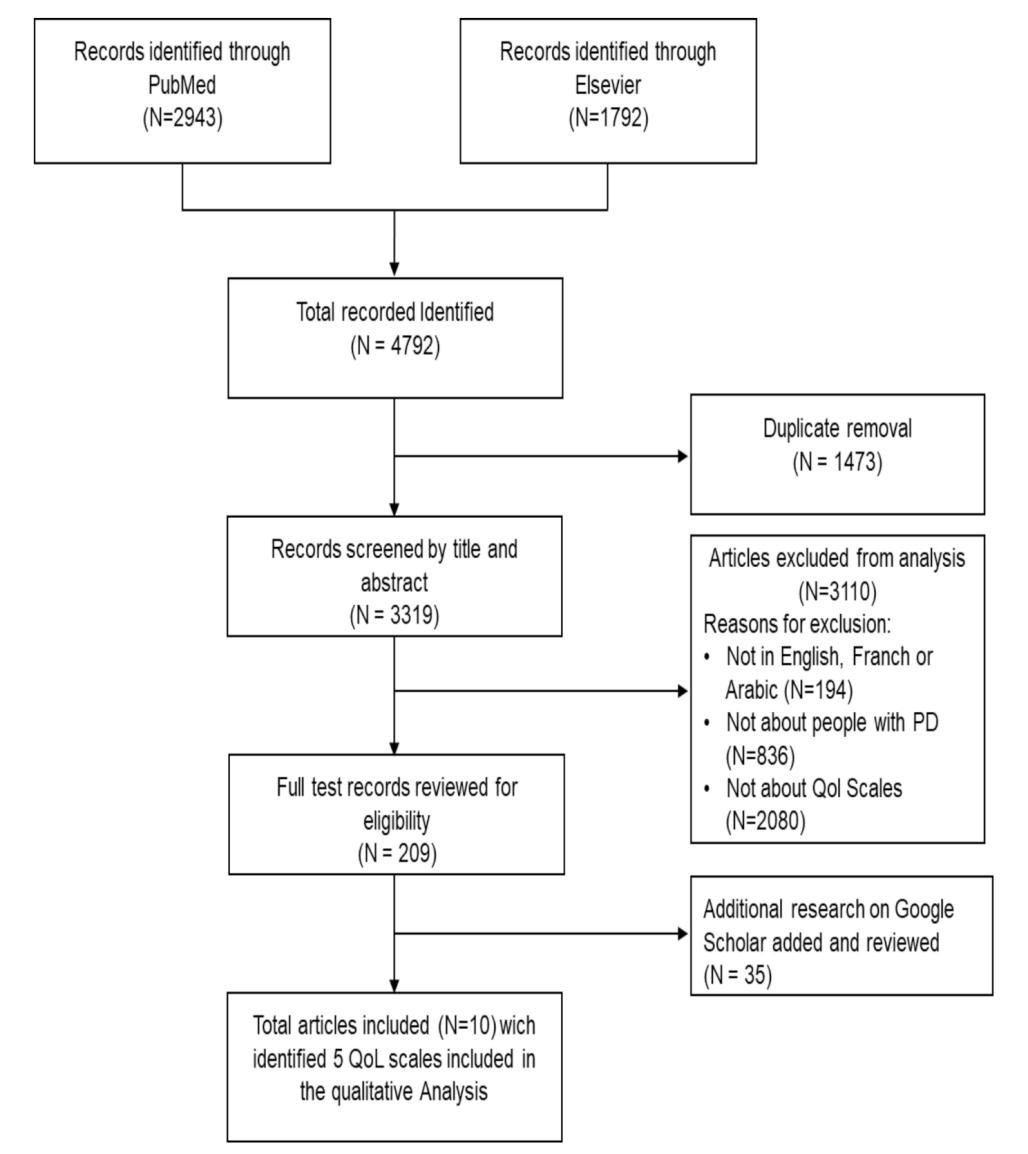
PRISMA-ScR flow diagram of the study selection process

A total of 4792 abstracts were identified from Medline (2943), Embase (1792) databases, and Google Scholar (35). After applying the inclusion/exclusion criteria, 10 studies including 5 scales were identified from the literature. The process for the selection of studies is illustrated in Fig. 1.

### Psychometric validation of the included scales: qualitative analysis and synthesis of results

As presented in Table 1, 5 HRQoL scales were included in this study. From these 5 scales, we identified 3 generic scales, and 2 versions (Moroccan and Egyptian). The 39-item Parkinson’s Disease Questionnaire (PDQ-39) PD-specific HRQoL scale. Two scales were validated in Arabic before 2010 (Short Form Health Survey (SF-36), European QoL 5-level version (EQ-5D-5L)) and 3 between 2010 and 2020. All the scales were interculturally validated in multiple languages. The PDQ-39 scale is the most validated HRQoL scale in people with PD. It was validated in more than 50 languages in its long form and 8 languages in its short form. Generic Scales included in this study, including the SF-36, the EQ-5D-5L, and WHOQoL-Bref (The World Health Organisation Quality of Life Scale BREF) were also validated in multiple languages, and Arabic validation of those scales is available [7, 46–50].

To summarise, the only PD-specific HRQoL scale that has been validated in Arabic is the PDQ-39. Generic scales have been used in PD populations without having been validated specifically for this group.

**Table 1:** HRQoL scales used in people with PD in Arabic: general characteristics and simplified quality criteria analysis

Name	Language	Preliminary hypothesis	Intended population	Items Nbr.	Item Identification	Nbr. of dimensions	Measure (Score)	Nbr. of patients	Nbr. of languages	Generic(G)/ Specific (S)	Validity	Sensitivity	Responsiveness	Ease of Use
PDQ-39 [51]	Arabic Egypt	+	++	39	0/++/++	8	0 to 100	97	50	S	+/++/-	++/+	+	+
PDQ-39 [52]	Arabic Morrocco	+	++	39	0/++/++	8	0 to 100	60	50	S	+/++/+	++/+	+	+
EQ-5D-5L [46]	Arabic Jordan	+	0	5	0/+/0	5	EQ-VAS 40 cm from 0 to 1	77	150	G	0/+/+	0/0		+
SF-36 [53]	Arabic Saoudi Arabia (KSA)	+	0	36	0/+/0	8	0 and 100	446	29	G	0/+/+	0/0	-	+
WHOQOL-BREF [54]	Arabic Kuwait	+	0	24	+/+/0	4	4–20 scores converted into a 0-100%	3303	19	G	0/+/+	0/0	-	+

The 39-item Parkinson’s Disease Questionnaire (PDQ-39); European QoL 5-level version (EQ-5D-5L); Short Form Health Survey (SF-36); The World Health Organisation Quality of Life Scale (WHOQOL-100); The World Health Organisation Quality of Life Scale BREF (WHOQOL-BREF).

GP General population, NR: not reported Rating applied in primary analysis grid: ++ = High; + = Medium; – = Low; 0 = Not reported

### Psychometric validation of the included scales: quantitative analysis and synthesis of results

HRQoL scales are used worldwide and, consequently, validated in several languages. Almost all scales evaluate dimensions such as health in general and physical and mental health, social relationships, activities of daily living, and leisure activities. Five scales were reviewed by the authors based on the established criteria (see supplementary Table 4). A further quantitative assessment of the 5 HRQoL scales is provided in Table 2. And all the details of the validation process of each scale is available (see supplementary Table 4).

The PDQ-39 scale, was initially developed by Jenkinson in 1995, then translated and validated in more than 50 languages is available in two Arabic versions. The PDQ-39 is the most used scale in the world. It is composed of 39 items with 8 dimensions, namely mobility (10-items), activities of daily living (6-items), emotional well-being (6-items), stigma (4-items), social support (3-items), cognition (4-items), communication (3-items), and bodily discomfort (3-items). In the Egyptian-Arabic adaptation of the PDQ-39, the study briefly described the translation process for the Arabic population. Reliability tests were not carried out [51]. For the Moroccan-Arabic adaptation of the PDQ-39, the study did not describe the translation process. Nevertheless, a high Cronbach’s alpha score was found in the reliability assessment, and a strong correlation was found in the interclass correlation analysis [52].

The SF-36 was translated and validated in Arabic as part of a study conducted in Saudi Arabia. This study included both the original English version and the translated Arabic version. Reliability assessment, including Cronbach’s alpha and test-retest reliability, was carried out and found to be acceptable, although the translation process was not described in detail [53].

The WHOQOL-Bref showed significant structural integrity in a previous cross-cultural validation study conducted in Arabic-speaking populations. In this study, the scale demonstrated acceptable construct validity, a good Cronbach’s alpha coefficient (between 0.69 and 0.93) coefficient and a highly significant test-retest interclass correlation (ICC values ​​was 0.95) [54].

The EQ-5D scale, which underwent intercultural validation in Jordan in Arabic, showed an acceptable Cronbach’s alpha coefficient (0.75) and a Cohen’s κ for test-retest reliability that ranged from 0.48 to 1.0. In addition, the EQ-VAS showed a significant correlation with age and SF-36 scores (*p* < .001) [46].

These generic scales have been validated in Arabic and used for people with PD, but have not been validated in this population. Table 2 provides a quantitative assessment of the five scales included. The maximum possible score is 42, which can be achieved if the study includes high psychometric validation and comprehensive information about the scale and its translations. The results in this table highlight the lack of evidence for validated scales in Arabic. The PDQ-39 score appears to be higher than generic scales. However, the overall scores remain low. This underlines the need for further studies to be conducted to validate these scales in an appropriate way.

**Table 2:** Quantitative analysis of *scale* development criteria and psychometric evaluation of HRQoL scales used in people with PD

Questionnaire	PDQ-39-Egypt	PDQ-39-Morrocco	WHOQOL-BRIEF-Kuwait	EQ-5D Jordan	SF-36-KSA
**Scale development**					
Pre-study hypothesis	1	1	1	1	1
Intended population	2	2	-1	-1	-1
Content validity	1	1	0	1	0
Conceptual definition/framework	0	0	0	0	1
Item identification	-1	0	0	1	1
Item selection	-1	0	0	-1	-1
Response	1	1	1	-1	1
Scoring	1	1	1	1	1
People with PD considered	2	2	-1	-1	-1
**Psychometric evaluation**					
Concurrent validity	-1	2	0	1	1
Predictive validity	-1	2	0	1	1
Convergent	1	0	1	0	0
Discriminant	1	0	1	1	0
Hypothesis testing	1	1	2	1	0
Group differences/Group Validity	0	0	1	0	0
Cross-cultural validity	0	0	0	0	0
Structural validity	0	2	1	0	0
Internal consistency	1	2	1	2	1
Test-retest reliability/agreement	0	0	2	1	1
Responsiveness	0	1	1	1	-1
Interpretation	1	1	1	1	1
TOTAL SCORE	9	19	12	9	6

Primary analysis grid rating: ++ = high; + = medium; - = low; 0 = not reported

Conversion of the rating into a numerical score: ++ = 2; + = 1; - = -1; 0 = not reported

Max possible score = 42

An explanation of the abbreviated names of the HRQOL scales is given under Table 1

## Discussion

Understanding the HRQoL of people with PD is essential to evaluate health interventions from a social and economic perspective properly. Ten studies assessing the HRQoL of people with PD through 3 generics, and 1 PD-specific HRQoL scale interculturally validated in two different Arab counties were found in this scoping review.

A systematic review on cross-cultural adaptation in Arabic-speaking populations was conducted in 2013, which identified generic HRQoL scales such as the SF-36, WHOQoL and EQ-5D scales that have been validated in different Arabic countries [41]. The conclusion of this review was that research on HRQoL assessment in Arab countries is limited and that, there is a clear need for further research on the performance of Arabic versions of these measures and their measurement properties, given the limited evidence on the performance of each identified scale. Since that time, additional scales have been validated in general or specific populations. For example, Arabic SF-36 validations are now available in Saudi Arabia, Tunisia, Lebanon and Morocco [47, 48, 55, 56]. While these validations have been carried out in general populations within their respective countries or in specific populations, they have not been carried out specifically in people with PD. However, the SF-36, EQ-5D and WHOQOL-Bref scales have been used in Arabic-speaking people with PD [57–60]. 

As predicted and shown in previous systematic reviews, the PDQ-39 is simultaneously the most used PD specific scale in the world and has the best psychometric properties. Those results are confirmed by a review made by Marinus in 2002, where he compared the validation method of PD-specific scales (PDQ, PDQL, PLQ, and PIMS) and proved that the PDQ-39 had the best psychometric proprieties [61]. Those results confirm those obtained by Martinez-Martin in 2011 [42] and also by Berardi in 2021 [43].

The Oxford University Innovation Clinical Outcome page pertaining to the Parkinson’s Disease Questionnaire (PDQ-39), noted that translated versions of the PDQ-39 exist in three countries (Egypt, Saudi Arabia, and Tunisia). However, to our knowledge, no validation studies have been conducted in Saudi Arabia, and Tunisia but, two different intercultural validation of the PDQ-39 in Arabic are available These two validating studies were conducted in Morocco and Egypt [51, 52]. Our results showed that compared to other PDQ-39 validated version [62–65], these two validation studies lack evidence. First, as the number of patients is less than 100 in both studies which is less than other PDQ-39 validation studies in the world. In addition, the translation process was not specified in the Moroccan version and, furthermore, a test-retest reliability test was not carried out in either study [51, 52]. In addition to a well-assessed psychometric properties, the development and use of HRQoL scales require appropriate methodology and studies that justify the choice of a scale [66, 67]. Scales that can show benefits and respond to study objectives are recommended in clinical research. Thus, disease-specific QoL measures designed to be more sensitive in assessing the impact of specific diseases or conditions on the affected population would be necessary [68].

While, most studies on HRQoL and PD have been performed in developed regions such as Europe and North America [69–71], data about HRQoL in South America, Africa, and the Arab world are scarce [72]. The impact of PD on individuals’ HRQoL can be significant even in regions with lower prevalence rates. The experience of PD and its impact on HRQoL can be influenced by factors such as access to healthcare, socioeconomic status, cultural beliefs and healthcare infrastructure.

Therefore, the need for culturally sensitive and validated scales to assess HRQoL remains critical, even though the prevalence of PD may be lower in certain regions. A scale including a linguistic validation that adapts preliminary translation and reflects cultural and linguistic differences (such as population demographics, genetics, lifestyle, and environmental influences) between diverse target populations is recommended [67, 73]. Through the use of such scales, researchers and health care professionals can gain insight into the unique challenges and needs of people with PD in different cultural contexts. Regardless of regional prevalence rates, this may lead to more effective interventions and improved patient outcomes. In this way, emphasising the importance of culturally specific tools will ensure that the assessment of HRQoL is comprehensive and meaningful across the diverse populations affected by PD.

Our scoping review acknowledges several limitations. The search was conducted in only two databases, with Google Scholar used as a supplementary tool to identify validation studies referenced in the initially included articles, rather than as the primary database. In addition, after testing different search strategies, we decided to search specifically for keywords in the title and abstract. While this approach ensured inclusion of relevant articles, it may have inadvertently excluded some relevant studies.

Moreover, the number of published studies in Arabic-speaking populations was limited. This discrepancy highlights a possible selection bias. Furthermore, relevant studies in other languages, such as Spanish, may have been excluded because this review only included studies published in English, French or Arabic. Additionally, the scoping review provides a broad overview of the existing literature, but does not include detailed data analysis or meta-analysis, which limits the depth of the analysis. Finally, as our inclusion criteria allowed for the inclusion of studies regardless of their quality, some low-quality studies were included, which may affect the overall reliability of the results.

Despite these limitations, this review has several strengths. It provides a broad exploration of the limited literature available in Arabic, particularly as, to our knowledge, no previous review has focused on HRQoL scales validated specifically for Arabic-speaking people with PD. The only existing reviews found were for the general population, making this scoping review a pioneering effort in understanding the use of HRQoL scales in Arabic-speaking people with PD. Additionally, this review identifies significant research gaps and limitations in the current literature, offering a foundation for future studies to address these gaps and improve the quality of research in this field. By mapping the available evidence, this scoping review pinpoints areas where further research is needed, ultimately guiding the development of more effective tools and interventions for Arabic-speaking people with PD.

To summarize, we recommend intercultural validation, which addresses cultural differences between the country where the scale was validated and the country in which it was translated. Currently, the PD Arabic population represents an unmet therapeutic need, as the number of patients in Arabic-speaking countries has increased significantly in recent years, as well as a highly personal, social, and economic burden for a large number of patients and their caregivers [74, 75].

## Conclusion

There is an array of HRQoL measuring scales for people with PD in research literature. However, only two validation studies of a specific HRQoL scale (PDQ-39) have been found in Arabic for people with PD and three generic scales validated for the general population but not specifically in people with PD, their suitability in the Arabic population and their psychometric properties are still limited, necessitating further research for clarification.

## Electronic supplementary material

Below is the link to the electronic supplementary material.

Supplementary Material 1

## Data Availability

The original contributions presented in the study are included in the article. Further inquiries can be directed to the corresponding author.
