# Supplementary material for: Psychometric properties of Arabic-translated-related quality of life scales for people with parkinson disease: a scoping review
Source: BMC Public Health. 2024 Sep 14;24:2505. doi: 10.1186/s12889-024-20002-0 (PMC11402201; doi:10.1186/s12889-024-20002-0)
Supplement: Supplementary file 1 — Supplementary Material 1 [file 12889_2024_20002_MOESM1_ESM.docx]

**Supplementary Materials**

Table 1: Search algorithm and results

| **Algorithms** | **Keywords** | **Pre-selected references**  **MEDILINE**  **Dec 2022** | **Pre-selected references**  **MEDILINE**  **Dec 2019** |
| --- | --- | --- | --- |
| #1 | Health related quality of life [tiab] | 56.893 | 42.066 |
| #2 | Quality of life [tiab] | 350.102 | 258.280 |
| #3 | Patient reported outcomes [tiab] | 21.572 | 11.490 |
| #4 | Measurement [tiab] OR measure [tiab] | 1.242.147 | 973.561 |
| #5 | Questionnaire [tiab] | 513.692 | 391.560 |
| #6 | Validation [tiab] | 286.730 | 197.555 |
| #7 | Development [tiab] | 2.622.195 | 2.088.387 |
| #8 | Test [tiab] | 1.758.359 | 1.430.609 |
| #9 | Scale [tiab] | 929.929 | 689.368 |
| #10 | Parkinson*[tiab] | 157.653 | 113.530 |
| #11 | Parkinson’s disease [tiab] | 142.781 | 80.314 |
| #12 | #1 or #2 or #3 | 363.764 | 265.271 |
| #13 | #4 OR #5 OR #6 OR #7 OR #8 OR #9 | 6.290.628 | 4.978.037 |
| #14 | #12 AND #13 | 169.297 | 113.532 |
| #15 | #10 OR #11 | 141.083 | 120.904 |
| #16 | #15 AND #14 | 3.070 | 2.530 |
| #17 | human filter | 2.943 | 2.209 |

Table 2: Simplified Quality Criteria Analysis Grid Questionnaires Used in people with PD

| 1. Study objectives and hypothesis | Is the rationale for the questionnaire explained, concepts explained and contextualised? | ++, +, -, 0 |
| --- | --- | --- |
| 2. Target population | Are people with PD involved?  If no, what is the other population? | ++, +, -, 0 |
| 3. Identifying and selecting data items | Has the pilot survey been subject to testing (quick or factor analysis, statistical justification of final items)?  Are they relevant to the target population?  In general: Is this study / target population relevant to people with PD? | ++, +, -, 0 |
| 4. Validity | Has internal validity been carried out?  Has the scale been previously compared with another scale?  If so, did the scale correlate with the results of the other scale? | ++, +, -, 0 |
| 5. Sensitivity | Can the Scale differentiate between people with and without PD?  between different stages of the disease and different levels of severity? | ++, +, -, 0 |
| 6. Responsiveness | Is the scale sensitive to clinical variation and trends through time? | ++, +, -, 0 |
| 7. Ease of use | Is the questionnaire convenient for patient administration/completion? (training required, time to complete, clarity of items, etc.) | ++, +, -, 0 |

Table 3: Quality criteria rating grid for people with PD

| Quality parameter | Definition | Rating |
| --- | --- | --- |
| Pre-study hypothesis | The pre-study reports the scale and the targeted population | ++ A clear explanation of the objective of the scale and of the population to be targeted  + Only one of the above  0 Not reported |
| Intended population | The level of evidence proving that the scale has been validated in people with PD. | ++ Intended population studied  + Partly studied, or small sample size (< 50 patients)  - Only generic |
| Content validity | The level to which the contents correspond to the hypotheses specified prior to the study. | ++ Content is relevant in people with PD.  + missing or irrelevant content  - Content not relevant t in people with PD  0 No people with PD involvement |
| Conceptual definition/framework | Conceptual definition/framework for the concept of interest has been given | ++ A conceptual definition/framework was included  0 No conceptual definition/framework |
| Item identification | Selecting relevant items to include in the pilot scale | ++ Comprehensive consulting with patients including focus groups or in-depth interviews and a literature review  + Minimum patient interviews, expert opinions and literature reviews  - No consultation with patients |
| Item selection | Identification of items for inclusion in the final instrument | ++ A pilot scale was developed and tested using Rasch or factor analysis. Statistical justification was included for the removal of items, and items with floor and ceiling effects were excluded and the level of missing data taken into account.  + Only part of the techniques listed above have been applied.  - No pilot test used or statistical evidence used to justify items included in the final Scale. |
| Response scale | Criteria used to evaluate the items | ++ Scale statistically justified with no major missing data, floor and ceiling effects, and ordered thresholds demonstrated by Rasch analysis.  + Some of the above, but not all  - Response scale methods not statistically justified |
| Scoring | Explaining how the Instrument is going to be scored | ++ Rasch scoring of statistically validated scaled responses  + Statistically justified response scale summary scoring  - Scoring system that is not defined, or scoring on a scale that is statistically inappropriate. |
| Views of people with PD considered | Percentage of people with PD included in item identification during the HRQoL scale development | ++ At least 50% of people with PD were involved in the item identification process  + Less than 50% people with PD were involved in the item identification Process  - No people with PD were involved in the item identification process |
| Psychometric evaluation |  |  |

Construct validity (→ the extent to which Scale scores match the hypothesis, based on available evidence of the construct) [1, 2]

| Quality parameters | Definition | Rating |
| --- | --- | --- |
| Convergent | The new measure is correlated with measures that, in theory, should be correlated with each other. | ++ Tested against appropriate measure, correlates between 0.3 and 0.9  + A questionable choice of measure, but correlation between 0.3 and 0.9  - Tested and correlates < 0.3 and > 0.9  0 Not available |
| Discriminant | The new measurement is not correlated with measures of characteristics other than the characteristic the measurement is intended to evaluate. | ++ Tested against appropriate measure, correlates <0.3  + A questionable choice of measure, but correlation <0.3  - Tested and correlates >0.3  0 Not available |
| Hypothesis testing | The measure is related in a way that would be expected to other measures among relevant groups | ++ Correlation with a scale that measures the same construct ≥ 0.75 or at least 50% of the results are consistent with the results are consistent with the hypothesis and correlate more with related constructs than with unrelated constructs.  + Only unrelated constructs were correlated with each other.  - Correlation <0.50 OR <75% of time as predicted, or correlates less with relevant measures than with irrelevant measures.  0 Not available |
| Group validity | The new measure will indicate significant variation between groups that are known to differ on this particular construct | ++ Significant differences between appropriate groups,  + A questionable choice of groups, but with significant differences between them  - Tested and nonsignificant difference between groups  0 Not available |
| Cross-cultural validity | The score of the items on a translated or culturally adapted scale adequately reflects the score of the items on the original version of the scale | + Confirmation of original factor structure or no significant DIF  - Non confirmation of original factor structure or significant DIF  0 Confirmation of original factor structure and not mentioned DIF |
| Structural validity | Using factor analysis to confirm the number of subscales present in a questionnaire, the extent to which the scores of an instrument adequately reflect the (uni)dimensionality of the construct being measured. | +Factors must explain at least 50% of the variance  - Factors explain <50% of the variance  0 Explained variance not mentioned |

Criterion validity (→ the extent to which instrument scores adequately reflect a gold standard) [1, 2]

| Quality parameters | Definition | Rating |
| --- | --- | --- |
| Concurrent validity | The new measure is correlating with scores on another measure of the same construct, or with a strongly related construct measured concurrently in the same subject. | ++ Tested against appropriate measure, correlates between 0.3 and 0.9  + A questionable choice of measure, but correlation between 0.3 and 0.9  - Tested and correlates < 0.3 and > 0.9  0 not available |
| Predictive validity | The test is an accurate predictor of the criterion for which the test is being used. | ++ Tested against appropriate measure, p<0.05  + A questionable choice of measure, but p<0.05  - Tested and p<0.05  0 Not available |
| Psychometric evaluation |  |  |

Reliability → The extent to which unchanging patients' scores are the same on repeated measurement under different conditions [1, 2] .

| Quality parameters | Definition | Rating |
| --- | --- | --- |
| Internal consistency | Systematic and random error in a patient's score not attributable to true changes in the measured construct, expressed as standard error of measurement (SEM). | ++ Cronbach’s alpha >0.70 and <0.90  - Cronbach’s alpha <0.70 and >0.90  0 Not available |
| Test-retest reliability if reported | The portion of total measurement variance due to true differences between patients, expressed as intraclass correlation coefficient (ICC) or Cohen's kappa. | ++ ICC/weighted kappa ≥0.70 or Pearson’s r≥0.80  0 Neither ICC/weighted kappa nor Pearson’s r determined  - ICC/weighted kappa <0.70 or Pearson’s r<0.80 |
| Responsiveness | The ability of the instrument to Identify clinical changes over time. | + Correlation with a measure of the same construct ≥ 0.50, or 75% or more of the scores agree with the hypotheses, or area under the curve (AUC) ≥ 0.70, and correlation with related constructs is more significant than with unrelated constructs.  0 Only correlations with an unrelated construct have been determined. constructs  - Correlated with a scale measuring the same construct |
| Interpretation | Score differences meaningful or not (assigning qualitative meanings to quantitative scores) (floor, ceiling effects, minimal important change, report) | ++ Normative data (i.e. mean scores and SD) and MID are given for a relevant population and the demographics of the test population are provided.  + Not all of the normative data are provided or no MID  - No normative data and no MID |

Table 4: Psychometric validation of included HRQoL Scales validated in people with PD in Arabic

| Name | Validation | | Reliability | | Article |
| --- | --- | --- | --- | --- | --- |
|  | Face Validity/ Content Validity | External structure validity evidence | Internal consistency | Reproducibility  (test-retest) |  |
| PDQ-39  Egypt | NR | Construct validity Multiple linear regression  All enrolled PD patients had impaired HRQoL with comparable values (scores between 40 and 50) and frequency (88.6–98.9%) in most dimensions, except for social support, cognition, and communication (had lower scores, indicating better HRQoL aspects)  86% of PD patients felt stigmatized, 56.8% of the cohort reported incomplete social support.  Females showed significantly more PDQ-SI, mobility, ADL, and bodily discomfort scores (p = 0.016, 0.001, 0.003, and 0.047, respectively).  The PDQ-SI score was inversely correlated with the patients’ age at enrolment (r = −0.220, p = 0.04), age of disease onset (r = −0.272, p = 0.01)  the PDQ-SI was positively correlated with H&Y (Off and on) disease stage (0.298, p = 0.003 and 0.006, (cognition) were the independent  . | Cronbach's alpha: between 0,66 and 0,95 | NR | [Shalash](https://pubmed.ncbi.nlm.nih.gov/?term=Shalash%20AS%5BAuthor%5D) 2018 [3] |
|  |  |  |  |  |  |
| PDQ-39  Morrocco | NA | Significant correlation coefficients using the 95% confidence interval and p <0.01.  The eight dimensions of PDQ-39 were correlated with HY and UPDRS.  Correlations were highest for the dimensions that measured the physical aspects  of patients’ health status (mobility and activities of daily living on PDQ-39)  Total score of PD correlated with H&Y, UPDRS and MMS (0.38, 0.3- and 0.86, respectively) with p < 0.001 | Cronbach’s alpha between 0.70 to 0.79 | NR | Ahmadou 2020 [4] |
|  |  |  |  |  |  |
| SF-36 KSA vs US-English | Recall period 2 weeks | Correlation of physical functioning, Role limitation physical, Role limitation Emotional, vitality and social functioning significant with P<0.01, general health was correlated with P< a 0.0001 and no significant correlation for mental health and bodily pain | Cronbach’s alpha between 0.60 and 0.87 | ICC values ​​ranged from 0.29 bodily pain to 0.80 mental health | Coons 1998 [5] |
|  |  |  |  |  |  |
| WHOQOL-BREF  Kuwait | missing values sexual satisfaction (7.9%), For the rest: ranged from 0.06% to <1% for 18 items, and from 1% to 1.8% for 7 items | The mean (SD) scores for the items ranged from 3.39 (1.2) to 3.89 (0.99) for 23 items; it was 4.0 (1.1) for two items (mobility and transport), and 2.96 (0.98) for one item (negative feelings). With regard to the floor and ceiling effects, the total frequency of lowest scores was 4.7% (range, 2.2% to 8.9%), while the total frequency of highest scores was 24.9% (range, 6.8% to 42.2%). The comparable figures for the WHO report were 4.0% (range, 1.7% to 8.8%) and 17.5% (range, 10.1% to 35.2%), respectively.6 | Cronbach’s alpha between 0.69 and 0.93 | ICC values ​​was 0.95 | Ohaeri 2009  [6] |
|  |  |  |  |  |  |
| EQ-5D Jordan | Recall period: 3-weeks | Construct validity  EQ-VAS correlated with SF-36 and age (0.83 et 0.54) with p < 0.001  Subjects reporting moderate or extreme problems for EQ-5D dimensions had lower SF-36 scores than those without such problems. Similarly subjects reporting problems for EQ-5D mobility, self-care, usual activities or pain/discomfort dimensions had larger score reductions for SF-36 | Cronbach’s alpha: 0.75. | Cohen's κ for test-retest reliability ranged from 0.48 to 1.0. | Aburuz  2009 [4] |

NR Not Reported; Intra-class correlation coefficients (ICC); Visual analog scale (VAS); Spearman's rank correlation coefficient (r); HY Hoehn and Yahr; UPDRS: Unified Parkinson’s Disease Rating Scale; MMS: Mini-mental state

References

1. De Vet H. C. W, T.C.B., Mokkink L. B, KnolD. L (2011);Cambridge University Press, et al.

2. Gazzard, G., et al., *A Scoping Review of Quality of Life Questionnaires in Glaucoma Patients.* Journal of Glaucoma, 2021. **30**(8).

3. Shalash, A.S., et al., *Clinical Profile of Non-Motor Symptoms in Patients with Essential Tremor: Impact on Quality of Life and Age-Related Differences.* Tremor Other Hyperkinet Mov (N Y), 2019. **9**.

4. Aburuz, S., et al., *The validity and reliability of the Arabic version of the EQ-5D: a study from Jordan.* Ann Saudi Med, 2009. **29**(4): p. 304-8.

5. Coons, S.J., et al., *Reliability of an Arabic version of the RAND-36 Health Survey and its equivalence to the US-English version.* Med Care, 1998. **36**(3): p. 428-32.

6. Ohaeri, J.U. and A.W. Awadalla, *The reliability and validity of the short version of the WHO Quality of Life Instrument in an Arab general population.* Ann Saudi Med, 2009. **29**(2): p. 98-104.
